# Survival-associated alternative splicing signatures in non-small cell lung cancer

**DOI:** 10.18632/aging.102983

**Published:** 2020-04-13

**Authors:** Deze Zhao, Chuantao Zhang, Man Jiang, Yongjie Wang, Yu Liang, Li Wang, Kang Qin, Faisal UL Rehman, Xiaochun Zhang

**Affiliations:** 1 Department of Medical Oncology, the Affiliated Hospital of Qingdao University, Qingdao University, Qingdao 266003, China; 2 Department of Thoracic Surgery, The Affiliated Hospital of Qingdao University, Qingdao University, Qingdao 266003, China; 3 Cancer Institute, The Affiliated Hospital of Qingdao University, Qingdao University, Qingdao 266071, China

**Keywords:** alternative splicing (AS), TCGA, LUAD, LUSC

## Abstract

Alternative splicing (AS) is fundamental to transcriptome and proteome richness, and data from recent studies suggested a critical association between AS and oncogenic processes. To date, no systematic analysis has been conducted on AS from the perspective of different sexes and subtypes in non-small-cell lung cancer (NSCLC). Thus, we integrated the information of NSCLC patients from The Cancer Genome Atlas (TCGA) and evaluated AS profiles from the perspectives of sex and subtype. Eventually, a total of 813 and 1020 AS events were found to be significantly related to the overall survival (OS) of lung adenocarcinoma (LUAD) and lung squamous cell carcinoma (LUSC) patients. Four prognostic prediction models performed well at 1, 3, and 5 years, with an area under the receiver operating characteristic (ROC) curve (AUC) greater than 0.75. Notably, we explored the upstream splicing factors (SFs) and downstream regulatory mechanisms of the OS-associated AS events and verified four differentially expressed alternative splicing (DEAS) events via qPCR. These findings can provide important guidance for subsequent studies. In addition, we also constructed nomograms to facilitate early screening by clinicians and to determine patient outcomes in NSCLC.

## INTRODUCTION

Worldwide, lung cancer remains the leading cause of cancer morbidity and mortality, and it was estimated that there were 2.1 million new lung cancer patients and 1.8 million deaths in 2018 [1]. Approximately 85% of patients can be classified as the histological subtype known as non-small-cell lung cancer (NSCLC), of which lung adenocarcinoma (LUAD) and lung squamous cell carcinoma (LUSC) are the most prevalent subtypes [2]. At present, there is no effective method for early diagnosis, and treatment still involves applying systemic chemotherapy or targeting a certain gene. However, different transcriptions of the same gene could produce proteins with different structures, and increasing evidence has revealed that proteins generated by multiple alternative splicing (AS) play key roles in carcinogenesis (including limitless replication, tissue invasion and metastasis, sustained angiogenesis, and avoidance of immune destruction) [3–7] In recent decades, many breakthroughs have been made in the field of AS, which has attracted much attention for its clinical potential in cancer therapy [8–10].

Here, we used a comprehensive analytical approach to elucidate the unique role of AS in NSCLC. Thus, we rigorously screened NSCLC cohorts from The Cancer Genome Atlas (TCGA) database and explored systematic profiles of genome-wide AS events from different sex and subtype perspectives. Eventually, four powerful prognostic models and a splicing factor (SF)-AS network were constructed to reveal the mechanism of AS events affecting the prognosis of NSCLC. In addition, we also established a nomogram model to help clinicians detect early relapses and assess patient prognosis. This work has great guiding significance for experimental exploration and clinical research.

## RESULTS

### Integrated AS event profiles in TCGA NSCLC cohorts

**Figure 1:**
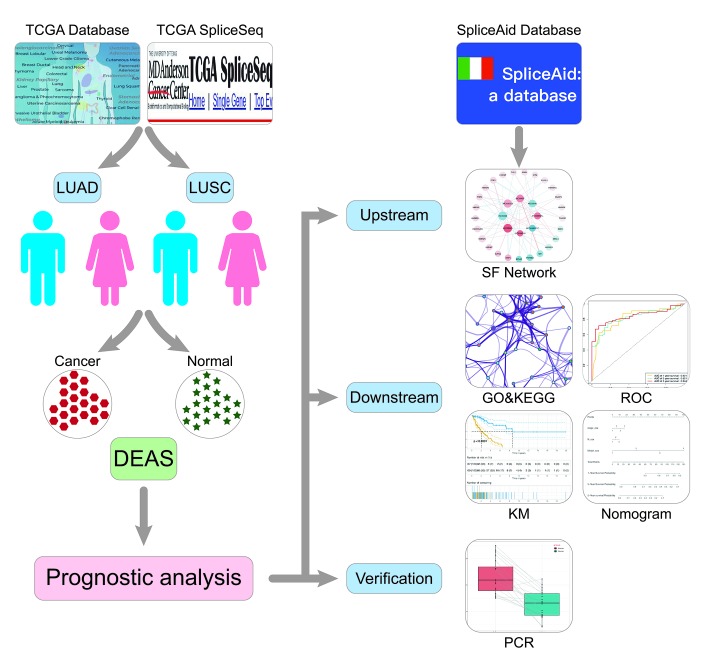
**Flowchart for profiling AS of NSCLC.** TCGA, The Cancer Genome Atlas; LUAD, lung adenocarcinoma; LUSC, lung squamous cell carcinoma; DEAS, differentially expressed alternative splicing; SF, splicing factor; KM, Kaplan Meier.

**Figure 2:**
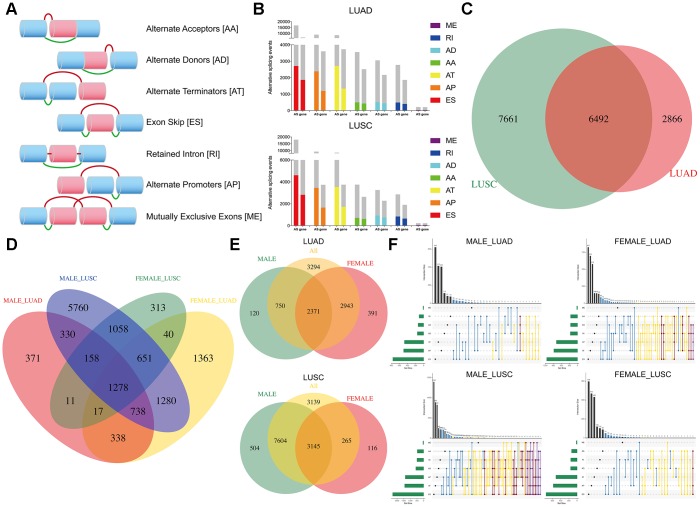
**Overview of DEAS events profiling in NSCLC cohorts.** (**A**) Schematic diagram of seven splicing pattern. (**B**) Seven types of AS events and corresponding parents’ genes. The gray bars represent the prognosis irrelevant AS events and related genes. The color bars represent the DEAS events and parent genes. (**C**–**E**) The Venn diagram compares DEAS in the four cohorts. (**F**) UpSet plot of intersections and aggregates among different types of DEAS events in NSCLC. One gene could have more than one type of OS-associated AS event.

The overview of the research design is presented in Figure 1. Finally, 491 LUAD patients and 473 LUSC patients were included in the analysis of integrated AS event signatures. In the LUAD group, a total of 43948 AS events from 10366 genes were detected, including 16793 Exon Skips (ESs) in 6618 genes, 8992 Alternate Promoters (APs) in 3605 genes, 8546 Alternate Terminators (ATs) in 3734 genes, 3559 Alternate Acceptors (AAs) in 2522 genes, 3057 Alternate Donors (ADs) in 2173 genes, 2781 Retained Introns (RIs) in 1866 genes, and 220 Mutually Exclusive Exons (MEs) in 214 genes. In the LUSC group, a total of 46020 AS events in 10557 genes were detected, including 18029 ESs in 6810 genes, 9301 APs in 3737 genes, 8578 ATs in 3748 genes, 3752 AAs in 2636 genes, 3263 ADs in 2278 genes, 2862 RIs in 1908 genes, and 235 MEs in 227 genes (Figure 2B). ES was the predominant component of AS, while ME was the least frequent type.

### Identification of differentially expressed AS (DEAS) events and survival analysis

By comparing the differences in AS events between tumor and normal tissues, we identified key events involved in tumorigenesis and prognosis. As shown in Figure 2B, AT occurs more frequently than AP in DEAS events but not in all AS events, which means that the AT type plays a more important role in tumor progression. Then, four Venn diagrams were generated to analyze the similarities and differences between these groups (Figure 2C–2E). We found that a number of AS events showed significant differences after distinguishing sex, and DEAS events in the female group were more common than those in the male group in LUAD, while the opposite trend was observed in LUSC. The distribution of DEAS events in each group is illustrated in the UpSet diagram. The male group of LUSC showed more sophisticated splicing, and many genes generated four or five DEAS events, which may affect tumorigenesis through compound effects (Figure 2F). The detailed characteristics of DEAS events are shown in Supplementary Table 3.

### Survival-associated AS events and functional enrichment analysis in NSCLC

In the univariate Cox regression analysis, 286 AS events in the male LUAD group, 582 AS events in the female LUAD group, 912 AS events in the male LUSC group and 113 AS events in female LUSC group were identified as candidate overall survival (OS)-associated AS events. The results of univariate Cox regression analysis are shown in Supplementary Table 4.

**Figure 3:**
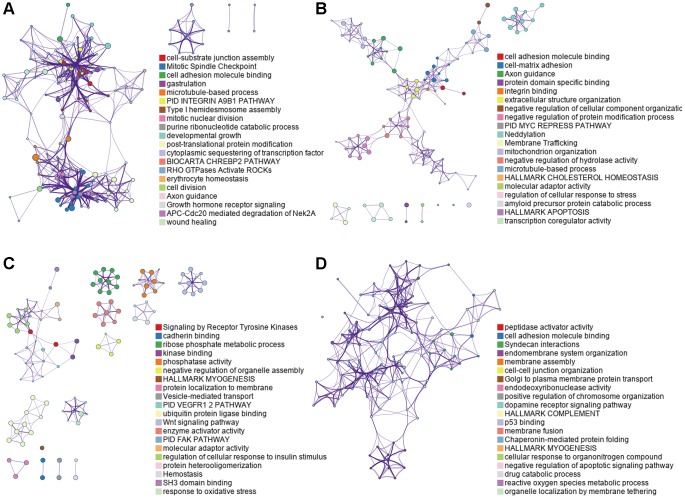
**Functional analyses on parent genes from OS-associated DEAS events in NCSLC, including GO and KEGG.** (**A**) LUAD_MALE group, (**B**) LUAD_FEMALE group, (**C**) LUSC_MALE group, (**D**) LUSC_FEMALE group.

Subsequently, in addition to the posttranslational modification level, we also attempted to clarify the underlying mechanisms of the OS-associated AS events. All corresponding genes were further assessed with functional and pathway enrichment analysis. The most significant enrichment results are shown in Figure 3. The commonly enriched terms in the four cohorts were “Hallmark epithelial-mesenchymal transition”, “cell adhesion molecule binding”, and “regulation of cell cycle process”, which were related to cell adhesion, migration, and division. In addition, many significant functional pathways were enriched in each group, which reflects their unique characteristics in tumorigenesis. The integrin A9B1 pathway, a multifunctional receptor that has important regulatory effects on the induction of pro-survival and pro-proliferative signaling cascades, was found to be significantly enriched in the male LUAD group. MYC repression, which plays an important role in cellular proliferation, differentiation, and apoptosis as well as cell cycle progression, was identified in the female LUAD group. The “VEGFR1/2 pathway” and “FAK pathway” were detected in the male LUSC group, and “p53 binding” was enriched in the female LUSC group. In summary, these analyses provide important clues for exploring the potential modification mechanisms of DEAS events in NSCLC and indicate that the incidence and subsequent treatment of the four subgroups may be different.

### Construction of prognostic models for NSCLC patients

**Figure 4:**
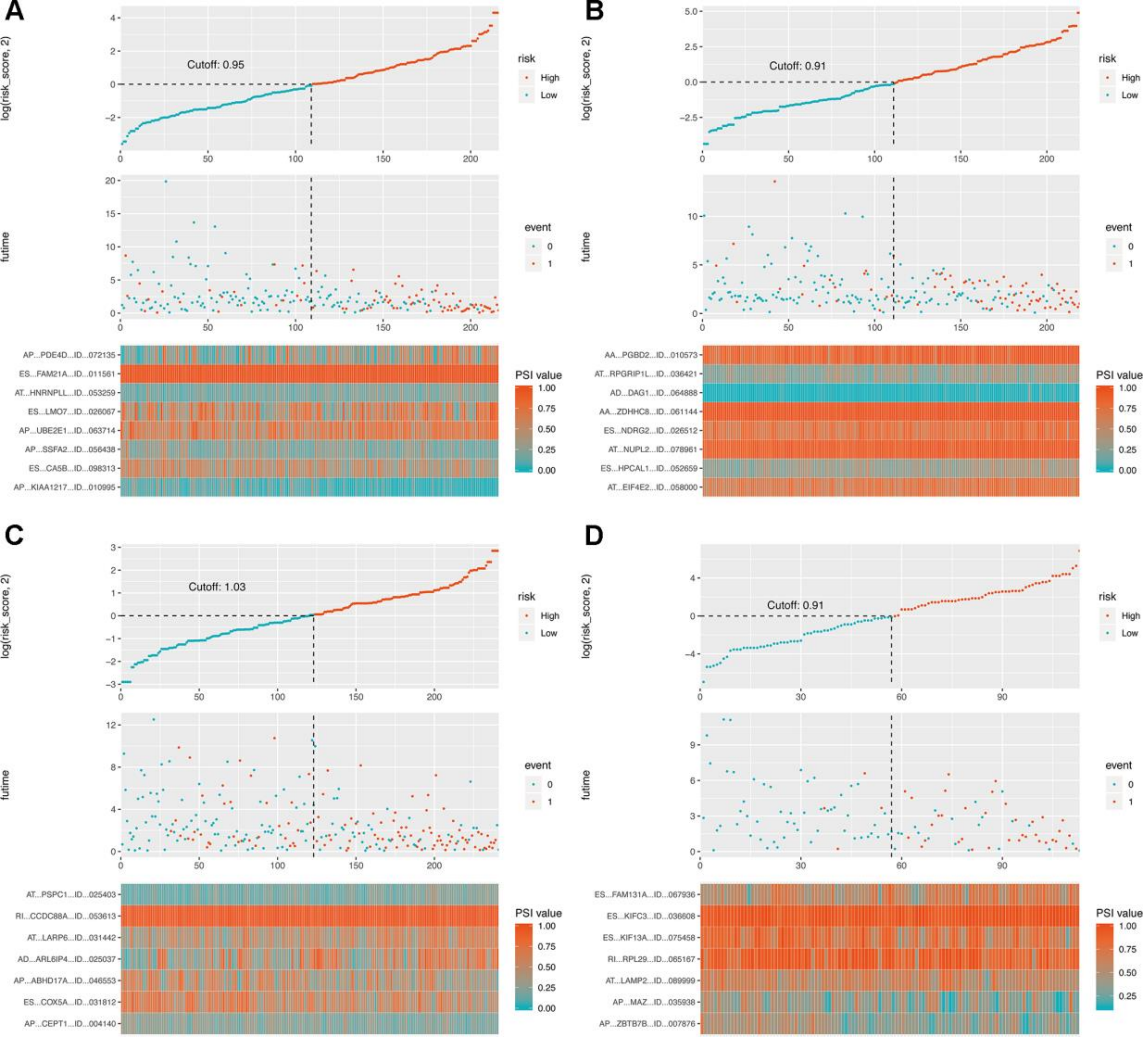
**Determination and analysis of the final prognostic models in four cohorts.** (**A**) LUAD_MALE group, (**B**) LUAD_FEMALE group, (**C**) LUSC_MALE group, (**D**) LUSC_FEMALE group. Patients were divided into high- and low-risk subgroups based on the median cut of risk score calculated separately. The upper part of each assembly indicates distribution of patients’ survival status and survival times ranked by risk score, the middle part represents the risk score curve, and the bottom heatmap displays splicing pattern of the AS from final prognostic models. Color transition from blue to red indicates the increasing PSI score of corresponding AS event from low to high.

**Figure 5:**
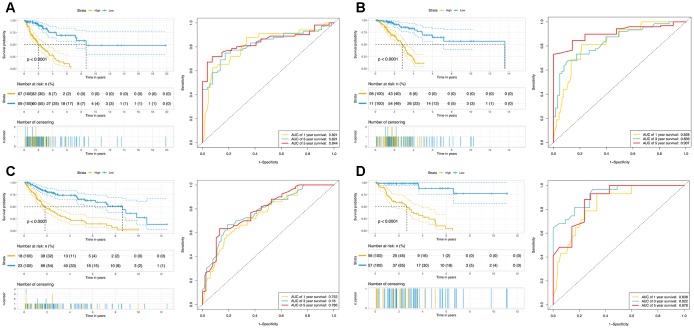
**The prognostic analysis of DEAS events in NSCLC.** (**A**) LUAD_MALE group, (**B**) LUAD_FEMALE group, (**C**) LUSC_MALE group, (**D**) LUSC_FEMALE group. The left plot is the K-M plot of prognostic models constructed with OS-related DEAS events for NSCLC patients. The right plot is ROC curves with calculated AUCs of prognostic models constructed with OS-related DEAS events.

The OS-related DEAS events in each cohort were used for lasso analysis to minimize the residual sum of squares plus a penalty term, and the results were used in multivariate Cox regression by the forward stepwise method. Finally, four composite models were constructed, and Kaplan-Meier (K-M) survival analysis of the composite models showed considerable power in distinguishing good or poor outcomes between the two subgroups (p < 0.0001, Figure 4, Supplementary Figure 2). The ability of the final models to classify patient survival, risk scores and splicing patterns is illustrated in Figure 5. Moreover, receiver operating characteristic (ROC) curves from 1 to 5 years were generated with the areas under the curve (AUCs) calculated. As shown in Figure 4, the final composite models exhibited strong predictive power, and the AUCs of each group were all over 0.75 from 1 to 5 years. In addition, the details of the composite models are shown are shown in Supplementary Table 5.

### AS-clinicopathological nomogram

**Figure 6:**
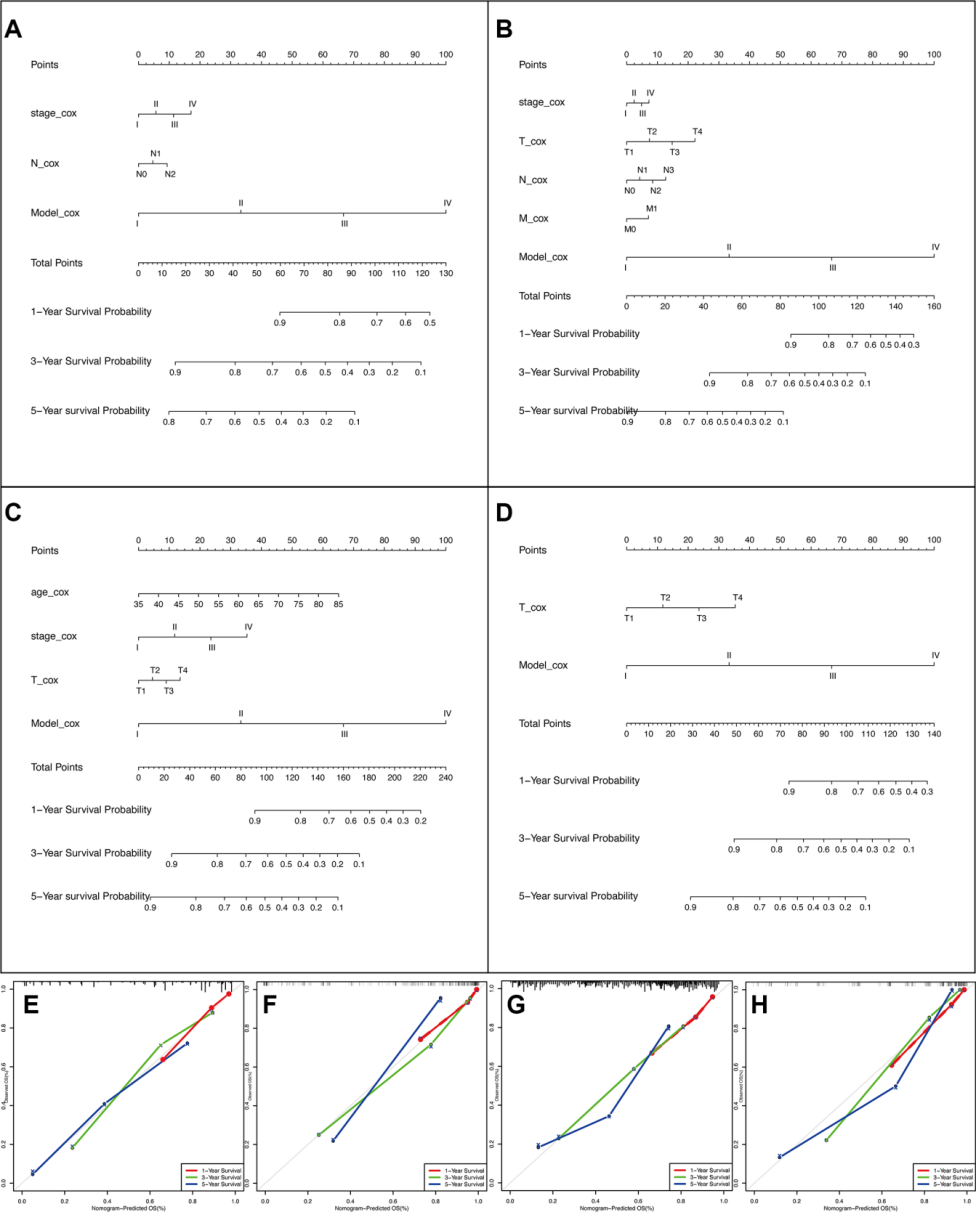
**The AS-clinicopathologic nomogram for prediction on survival probability in patients with NSCLC.** (**A**–**D**) Development of AS-clinicopathologic nomogram for predicting 1-, 3-, and 5-years OS for LUAD_MALE group, LUAD_FEMALE group, LUSC_MALE group, and LUSC_FEMALE group, with the final AS signature and independent prognostic factors. (**E**–**H**) Calibration plot of the AS-clinicopathologic nomogram in terms of agreement between nomogram-predicted and observed 1-, 3-, and 5-years outcomes in four cohorts. The actual performances of our model are shown by green, blue, and red lines. And the silver line of 45° represents the ideal performance.

To expand the application of AS events, we tried to establish nomograms to connect AS events with clinical treatment. Ultimately, the clinicopathological variables included in the model included age, pathological stage, T stage, N stage, M stage, and the final composite models (Figure 6A–6D). It is worth mentioning that we divided the risk score of the AS model into four levels to ensure the practicality of the nomogram. In addition, the calibration curve of the nomograms showed good uniformity between the forecast and the actual prognosis (Figure 6E–6H). Moreover, the concordance index (C-index) for OS prediction was 0.777 (95% confidence interval (CI): 0.748-0.806) in the male LUAD group, 0.827 (95%CI: 0.796-0.858) in the female LUAD group, 0.729 (95%CI: 0.704-0.754) in the male LUSC group, and 0.843 (95%CI: 0.813-0.873) in the female LUSC group. In summary, the results demonstrated that the constructed nomogram had great potential for application in clinical practice.

### Potential differentially expressed SF (DESF) regulatory network construction

**Figure 7:**
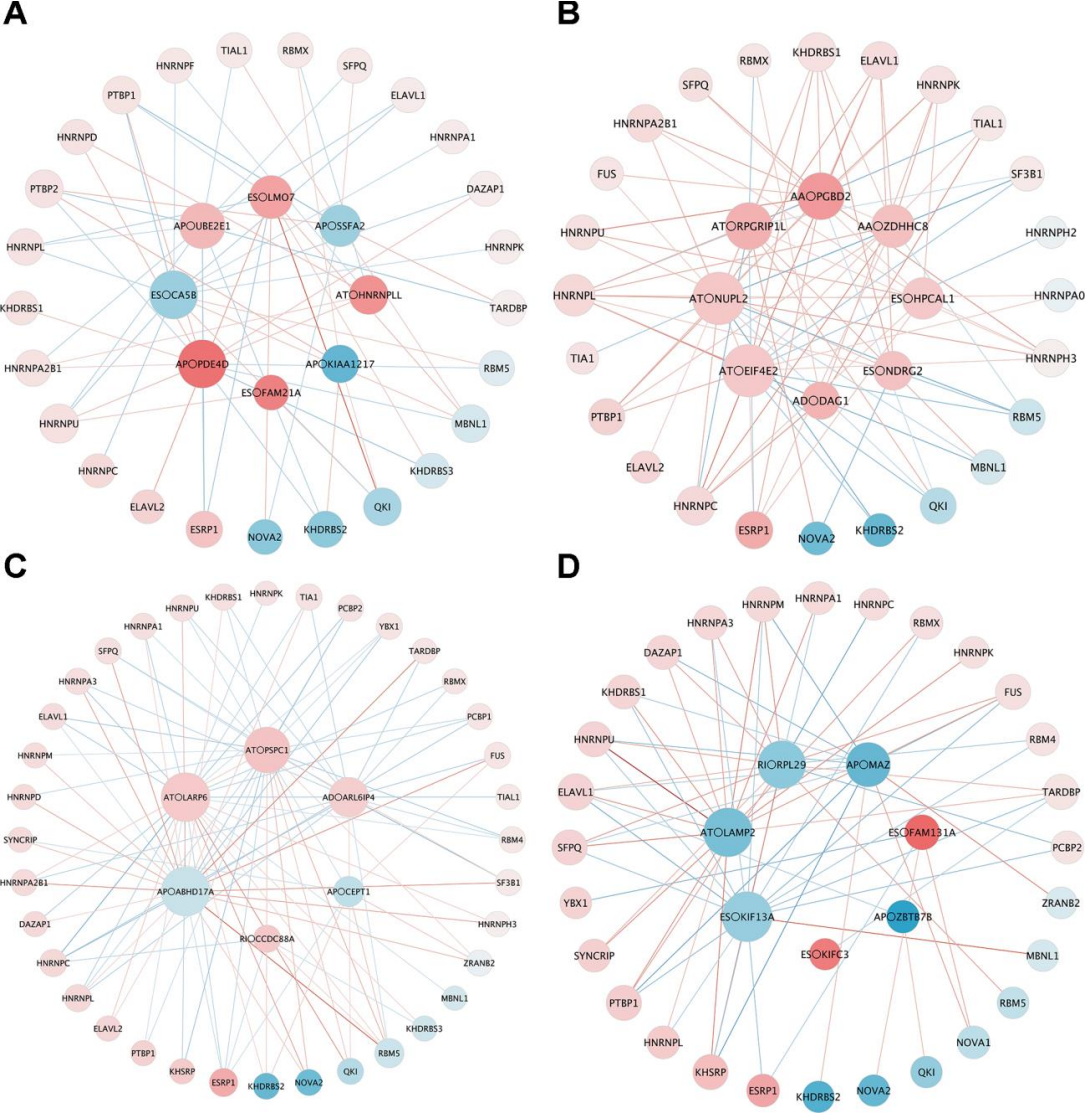
**The correlation network of DESF and OS-related DEAS.** (**A**) LUAD_MALE group, (**B**) LUAD_FEMALE group, (**C**) LUSC_MALE group, (**D**) LUSC_FEMALE group. The size indicates the degree of the point in the network, and the intensity of the color indicates the strength of its characteristics. For DESFs, the peripheral red dots indicate upregulation (log fold change (FC) > 1), while the blue dots indicate downregulation (logFC < 1). For DEAS events, the central red dots indicate poor prognosis (hazard ratio (HR) > 1), while the blue dots indicate better clinical outcomes (HR < 1).

To explore the upstream mechanism of AS regulation, we analyzed the RNA sequencing data of SFs from the TCGA database. A total of 26, 27, 37 and 31 SFs were identified as significantly different between tumor and normal tissues in the male LUAD, female LUAD, male LUSC, and female LUSC groups, respectively. Subsequently, correlation analysis was conducted between DESFs and DEAS events, and the results are shown in Figure 7 (only significant correlations with p < 0.05 are presented). The size indicates the degree of the point in the network, and the intensity of the color indicates the strength of its characteristics. For DESFs, the peripheral red dots indicate upregulation (log fold change (FC) > 1), while the blue dots indicate downregulation (logFC < 1). For DEAS events, the central red dots indicate poor prognosis (hazard ratio (HR) > 1), while the blue dots indicate better clinical outcomes (HR < 1).

### Verification of DEAS events in tissue by real-time quantitative PCR

To verify the accuracy of the bioinformatics analysis, we collected pairs of tissue samples for further verification, including 20 pairs of tissue samples in the male LUAD, 20 pairs of tissue samples in the female LUAD group, 10 pairs of tissue samples in the male LUSC group, and 10 pairs of tissue samples in the female LUSC group.

**Figure 8:**
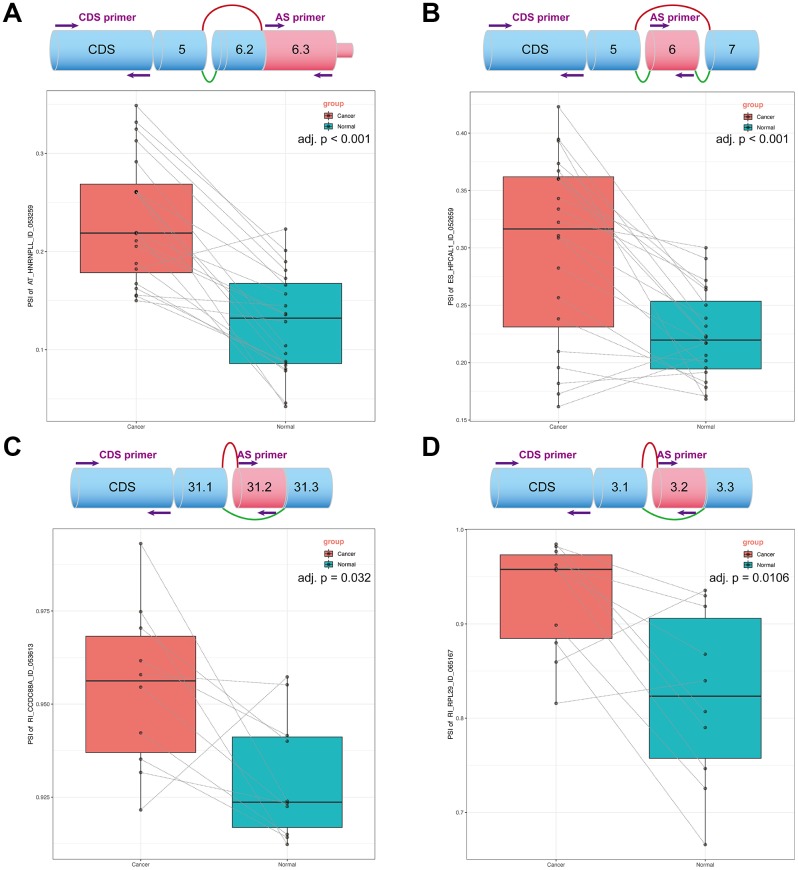
**OS-related DEAS events expression in NSCLC.** (**A**) The top plot demonstrated the splicing pattern of AT〇HNRNPLL〇ID〇053259. The bottom plot displays the PSI of AT〇HNRNPLL in cancer and normal tissues. (**B**) The top plot demonstrated the splicing pattern of ES〇HPCAL1〇ID〇052659. The bottom plot displays the PSI of ES〇HPCAL1 in cancer and normal tissues. (**C**) The top plot demonstrated the splicing pattern of RI〇CCDC88A〇ID〇053613. The bottom plot displays the PSI of RI〇CCDC88A in cancer and normal tissues. (**D**) The top plot demonstrated the splicing pattern of RI〇RPL29〇ID〇065167. The bottom plot displays the PSI of RI〇RPL29 in cancer and normal tissues. In the schematic diagram, green lines represent transcripts before splicing and the red lines represent transcripts after splicing.

We reviewed related studies on the parent genes of all splicing events, and four DEAS events from each AS model were eventually selected for further verification. Two primers for each gene were designed to keep the experimental methods consistent. One primer was located on the splicing sequence of the DEAS events, and another was located on the CDS sequence of all transcripts. We used qPCR to obtain the expression of splicing events and CD regions of each gene, and the ratio of the two expressions is the percent spliced in (PSI) value; moreover, the ES type is -1 of this PSI value. The detailed data obtained from the qPCR is shown in Supplementary Table 6. Box plots were generated to illustrate the qPCR results (Figure 8). The ratio of these four DEAS events was significantly upregulated in tumor tissue, which indicated that an increase in these DEAS events would affect the generation of tumors. Importantly, these findings provide important guidance for more detailed functional tests that we will conduct next.

## DISCUSSION

The carcinogenesis of NSCLC is an intricate regulatory network. Thus, gathering diverse biomarkers and establishing a model is an effective way to predict tumor prognosis compared to using a single clinical indicator. In the past decade, numerous studies have focused on integrating genome-wide prognostic biomarkers to improve the prognosis and diagnosis of NSCLC. However, most of these studies were limited to transcription level analyses and utilized mRNAs, long noncoding RNAs or microRNAs for prediction models [11, 12].

AS is a posttranslational modification process that produces multiple mRNA isoforms with different regulatory abilities from a single gene. Cancer cells typically utilize the diversity of AS to generate isoform switches that contribute to carcinoma proliferation, invasion, metastasis, apoptosis, and drug resistance [3, 5, 13]. Recent evidence suggests that specific AS dysregulation has an important impact on the development and prognosis of NSCLC, and more than 12 splice variants have been suggested to be associated with lung cancer progression and/or response to therapies [14]. For instance, the VEGF_xxx_ and VEGF_xxxb_ families encode splice variants of VEGF-A that differ only at the level of six amino acids in their C-terminal part. Boudria (2019) found that a high VEGF_165b_/VEGF_165_ ratio was related to lymph node metastasis. VEGF_165b_ could promote the proliferation and invasiveness of lung tumor cells through a VEGFR/β1 integrin loop. Notably, bevacizumab, as a common VEGF inhibitor used for the treatment of LUAD patients, could increase the expression of VEGF_165b_ and activate the invasive VEGFR/β1 integrin loop [15]. In addition, MET is a high-affinity receptor tyrosine kinase (RTK) that can initiate several pathways promoting cell proliferation, survival, and metastasis. Joanna (2018) demonstrated that MET Exon 14 splice site mutations defined unique molecular subgroups of NSCLC with poor prognosis, and MET inhibition might benefit this specific subgroup of patients [16]. These studies proved that specific RNA splice variants could be cancer biomarkers or useful tools for the prediction of patient outcomes. Therefore, we performed a systematic analysis to clarify the role that splice variants may play in the carcinogenesis of NSCLC.

In addition, previous studies have reported that sex was one possible factor affecting cancer survival [17, 18], and female sex was an independent favorable prognostic factor for lung cancer [19, 20]. In our work, we first attempted to evaluate AS profiles from different sex and subtype perspectives. The results demonstrated that the OS-associated DEAS event profiles had significant individual variations among the four groups, and only a few AS events participated in the OS of multiple subtypes. Subsequently, we constructed multivariate Cox regression models for each subgroup, and the final composite models displayed a great discrimination ability. In addition, the OS-related DEAS events filtered by multivariate analysis could also be recognized as an independent prognostic indicator, and these DEAS events could be used in subsequent studies on the effect of different ratios of transcripts on tumorigenesis and prognosis.

Nomogram is a user-friendly graphical composite model to predict the likelihood of an event occurring, such as recurrence, based on the individual profiles of the patient. Currently, many nomograms have been proven to be effective in lung cancer, such as the EGFR mutation model and noncytotoxic chemosensitizer model [21, 22]. To make our prognostic models achieve a more reliable and valuable prediction efficacy in clinical practice, the prognostic nomogram, consisting of age, pathological stage, T stage, N stage, M stage and the final composite models, was developed for assessing the individualized survival risk of patients and showed satisfactory discrimination.

Moreover, we performed functional enrichment analysis to explore the underlying mechanisms of splice-associated carcinogenesis. The Gene Ontology (GO) and Kyoto Encyclopedia of Genes and Genomes (KEGG) analyses suggested that the majority of enrichment pathways varied among the different subtypes. It is generally believed that disordered AS events are produced by a few SFs, which can identify splicing information, bind to the pre-mRNA sequence and subsequently produce mature transcripts through coordinated interactions. To explore the upstream mechanisms of AS regulation, we constructed splicing correlation networks between the DESFs and DEAS events. The discovery of these potential pathways and upstream DESFs provides important guidance for the subsequent exploration of the functional mechanisms of these DEAS events.

HNRNPLL, an RNA-binding protein that participates in mRNA splicing and stability, was identified as a colorectal cancer metastasis suppressor. It could bind to CD44 pre-mRNAs and inhibit the epithelial-mesenchymal transition (EMT) of tumor cells [23]. Interestingly, AT_HNRNPLL was a risk hazard (HR > 1) in our prognostic model and was indeed upregulated in tumors according to our validation. Here, we speculate that the occurrence of AT events causes HNRNPLL to terminate early and lose its original function.

HPCAL1, a neuronal calcium sensor protein, was found to be upregulated by Ca^2+^ in glioblastoma (GBM) tissues and cells. It could facilitate the proliferation of GBM by activating the Wnt/β-catenin pathway and promoting the expression of the *c-Myc* gene [24]. In our research, the upregulation of ES_HPCAL1 negatively affected tumor prognosis (HR > 1), but the MYC repression pathway was significantly enriched in the functional analysis. The occurrence of this phenomenon may be a protective measure of organisms against tumors.

CCDC88A, a substrate of the threonine/serine kinase Akt, was identified as a participator in the migration and invasiveness of pancreatic ductal adenocarcinoma (PDAC) cells. It could increase the phosphorylation of Src and ERK1/2 and decrease the phosphorylation of AMPK1 in PDAC cells [25]. In our research, RI_CCDC88A, as a risk factor (HR > 1), was found to be upregulated in tumors. Apart from this pathway, some other classically important pathways are enriched, such as the VEGFR1/2, FAK and Wnt pathways.

RPL29, an important protein in protein synthesis, was upregulated in colon cancer cells. The resultant repression of HIP/RPL29 induces cell differentiation with the upregulation of p21 and p53 [26]. According to our analysis, as a protective factor (HR < 1), RI_ RPL29 was upregulated in tumors, and the p53 binding pathway was significantly enriched. The appearance of the RI event might change the structure of RPL29, resulting in the loss of its original function.

Nevertheless, our exploration of the mechanisms is not deep enough, but these findings provide important guidance for our future work.

In addition, the patients we selected were from a single cohort of a single database, and there were no other cohorts available, especially cohorts with prospective data that could be used to verify that the nomogram presented here is repeatable. Due to limited published data, the analysis of clinical and pathological features was not thorough, which may affect our results. Nevertheless, this in-depth analysis of splicing patterns provides new molecular changes and potential drug targets. More basic mechanistic research and large-scale statistical analyses are necessary to validate the current composite models in the future.

In summary, this study performed a systematic analysis on prognostic splicing events in NSCLC from sex and subtype perspectives. Then, we explored the upstream regulatory factors and downstream regulatory mechanisms of the OS-related AS events found in our study. More importantly, we constructed a well-executed nomogram that combines clinicopathological variables with four composite models. The final AS events obtained through screening may play an important role in tumorigenesis and deserve further study as molecular diagnostic biomarkers and therapeutic targets.

## MATERIALS AND METHODS

### Data collection for AS events

The RNA transcriptome profiles of the LUAD and LUSC cohorts were retrieved from the TCGA data portal (https://tcga-data.nci.nih.gov/). AS events were obtained from TCGA SpliceSeq. The PSI value, which represents the ratio between reads including or excluding exons, was calculated for the following splice events in the four cohorts: AA, AD, AP, AT, ES, ME, and RI (Figure 2A) [27]. To obtain a reliable dataset of splicing events, a strict screening filter was set with a sample percentage of PSI values of not less than 75. The raw data are presented in Supplementary Tables 1 and 2. The screening criteria were as follows: (1) definite histological diagnosis of NSCLC; (2) integrated and clear clinical variables, including age, pathological stage, and TNM stage; (3) OS after the initial pathological diagnosis of more than 30 days; and (4) patients with corresponding RNA-seq splicing variant data. As a result, 491 LUAD patients and 473 LUSC patients were eventually included in our analysis cohort.

Simultaneously, each splicing event was allocated a unique code consisting of the splicing type, gene symbol and ID number to ensure accuracy. For example, in the code “ES〇PLEKHN1〇ID〇000001”, ES indicates the splicing type, PLEKHN1 is the symbol of its parent gene and “ID〇000001” indicates the ID number of the splicing event.

### Identification of DEAS events.

The LUAD and LUSC cohorts were divided into two groups by sex, and paired samples of each group were screened out for further calculation. The limma package was used for differential analysis. Adjusted p value <0.05 was applied as the threshold to avoid missing significant changes. Then, Venn diagrams were generated to illustrate the differences among the four groups. In addition, an UpSet plot was constructed to demonstrate intersecting sets between the seven types of DEAS events [28].

### Survival, GO functional and KEGG pathway enrichment analyses of DEAS events

Each group was separated into two groups by the median PSI value. Then, univariate Cox regression was conducted to explore independent prognostic factors with p < 0.05.

Next, we selected the corresponding parent genes of the OS-associated DEAS events as candidates for GO and KEGG pathway enrichment analysis using Metascape [29]. P < 0.05 was statistically significant.

### Prognostic model construction

Then, the OS-associated DEAS events identified above were selected as candidates for lasso analysis (Supplementary Figure 1), and the results were used for further multivariate Cox regression via the forward stepwise method [30]. Afterward, risk scores were calculated based on each prognostic model, and the patients were separated into two subgroups by the median risk score. K-M survival analysis and dynamic time-dependent ROC curves were conducted to validate the predictive accuracy of the prognostic models. For these analyses, the survminer package (version 0.4.3), survivalROC package (version 1.0.3) and timeROC package (version 0.3) in R were used.

### AS-clinicopathological nomogram

Subsequently, the prognostic models along with the clinicopathological variables described above were used in the univariate Cox analysis, and the significant results were further used to develop a nomogram to estimate the individual survival probability of patients. Then, we plotted calibration curves and calculated the C-index to validate and quantify the discrimination ability of the scoring system.

### Construction of the potential SF-AS regulatory network

A total of 67 human SFs were retrieved from the SpliceAid 2 database [31]. The RNA sequencing data of the SFs were downloaded from the TCGA database and normalized by the DESeq2 package (version 1.22.2) [32]. Similarly, the differential analysis of SFs was conducted in four groups (with adj. p < 0.05). Subsequently, the correlation between the expression of DESFs and PSI values of OS-associated DEAS events was calculated by Pearson analysis. P < 0.05 was set as the cut-off value for these analyses. Finally, four potential SF-AS regulatory networks were generated based on the significant results of Spearman correlation analysis via Cytoscape (version 3.7.1).

### Real-time quantitative PCR to verify DEAS events

Real-time quantitative PCR was conducted to validate the selected OS-associated DEAS events. Total RNA from frozen tissues was isolated with TRIzol (Invitrogen, ThermoFisher, CA). The PrimeScript^TM^ RT Kit (TaKaRa, Otsu, Japan) was used to reverse transcribe the total RNA into cDNA. SYBR Premix EX-Taq^TM^ (TaKaRa, Otsu, Japan) was used for qPCR on an FTC-3000p PCR system (Funglyn Biotech, Shanghai, China). Relative gene expression was calculated by the comparative 2^−ΔΔCT^ method. The PCR primers used are listed in Supplementary Table 6.

## Supplementary Material

Supplementary Figures

Supplementary Table 1. PSI_LUAD_1

Supplementary Table 1. PSI_LUAD_2

Supplementary Table 2. PSI_LUSC_1

Supplementary Table 2. PSI_LUSC_2

Supplementary Table 3

Supplementary Table 4

Supplementary Table 5

Supplementary Table 6
