# Supplementary material for: Survival-associated alternative splicing signatures in non-small cell lung cancer
Source: Aging (Albany NY). 2020 Apr 13;12(7):5878–93. doi: 10.18632/aging.102983 (PMC7185095; doi:10.18632/aging.102983)
Supplement: Supplementary Figures [file aging-12-102983-s003..pdf]

SUPPLEMENTARY FIGURES

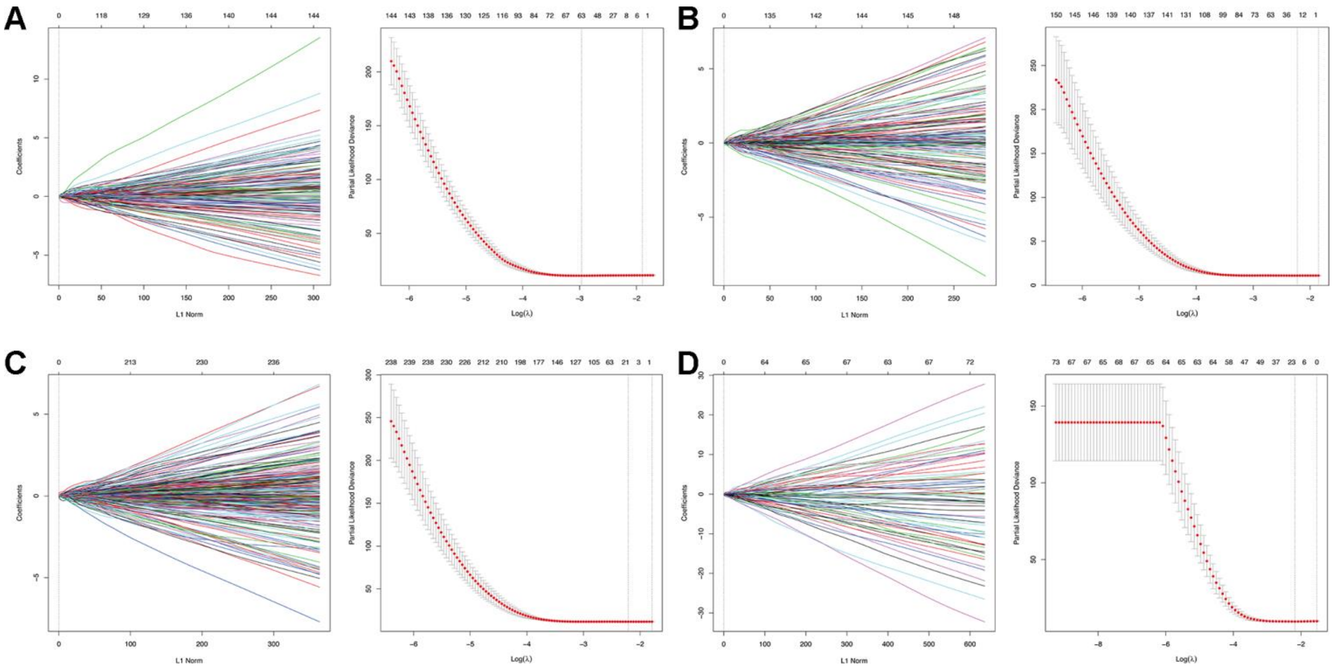

Supplementary Figure 1. LASSO analysis for OS-related DEAS events. (**A**) LUAD_MALE group, (**B**) LUAD_FEMALE group, (**C**) LUSC_MALE group, (**D**) LUSC_FEMALE group. In left plot, each curve showed an OS-related DEAS event; ten-fold cross-validation was used to calculate best lambda which leads to minimum mean cross-validated error. The right plot was the partial likelihood deviance of the LASSO coefficient profiles.

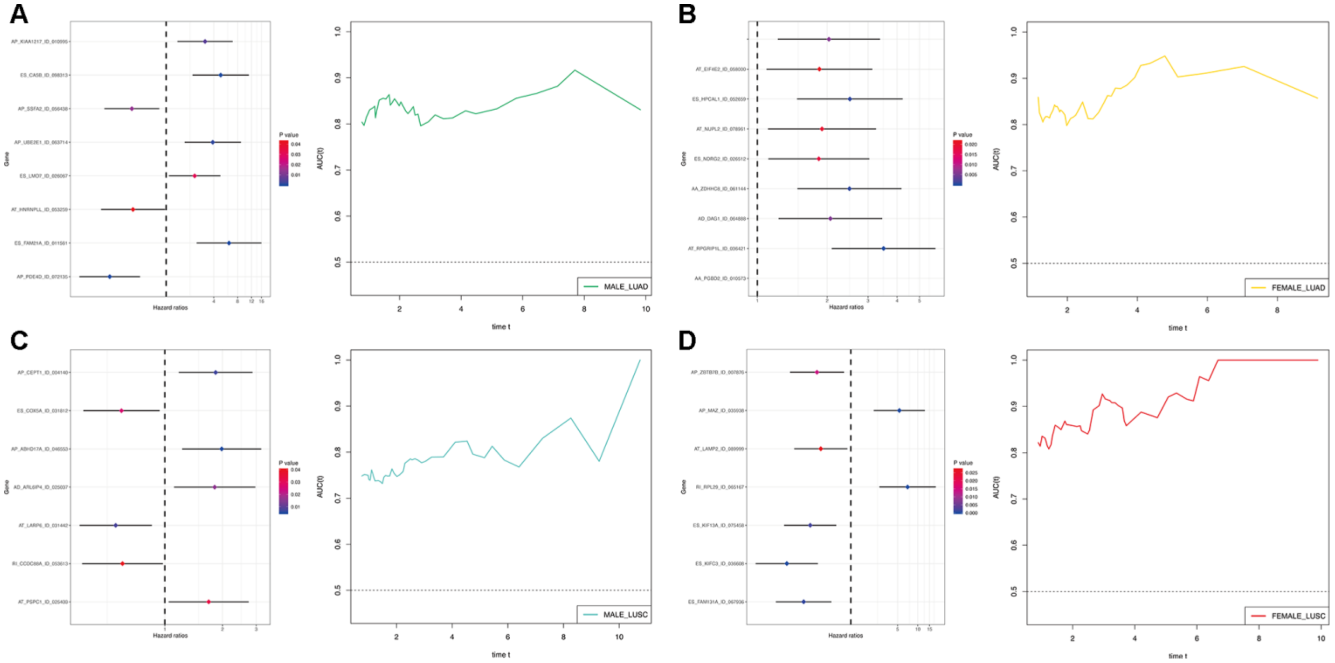

Supplementary Figure 2. The prognostic analysis of the final models in four cohorts. (**A**) LUAD_MALE group, (**B**) LUAD_FEMALE group, (**C**) LUSC_MALE group, (**D**) LUSC_FEMALE group. The left plot is the forest plot of prognostic models. p-values are indicated by the color scale on the side. The right plot is the curve of time-dependent AUCs versus time: AUC(t) versus t.
